# Supplementary material for: CXCR5 Signals Fine-Tune Dendritic Cell Transcription and Regulate TH2 Development
Source: Vaccines (Basel). 2025 Sep 3;13(9):943. doi: 10.3390/vaccines13090943 (PMC12474269; doi:10.3390/vaccines13090943)
Supplement: Supplementary file 1 [file vaccines-13-00943-s001.zip › vaccines-3791020_R1supp.pdf]

vaccines

MDPI

Article

CXCR5 Signals Fine-Tune Dendritic Cell Transcription and Regulate T_H2 Development

Supplemental Tables and Figure Legends

Table S1 related to Figures 4 and 5. RNA-seq analysis of msLN migratory cDC2 and cDC1 cells isolated from D8 *Hp*-infected 50:50 wildtype: *Cxcr5*$^{-/-}$ chimeric mice. 1:1 B6:*Cxcr5*$^{-/-}$ chimeric mice were generated (as in Fig. 1A) and infected with *Hp*. B6 (CD45.1^{+}) and *Cxcr5*$^{-/-}$ (CD45.2^{+}) cDC1 (CD103^{+}CD11b^{-}) and cDC2 (CD103^{+}CD11b^{+}) cells were sort-purified from d8 *Hp*-infected animals (as in Fig. S2A-E). RNA was isolated (samples derived from 3 independent experiments with 15 mice/group/experiment) and bulk RNA-sequencing was performed. Sequencing data was analyzed as described in Methods. Data reported as RPKM values for each expressed gene, which is defined as a gene with at least 3 reads per million in all samples of at least one group. log2FC values reported for (i) CD45.1^{+} B6 WT cDC1 cells over CD45.2^{+} *Cxcr5*$^{-/-}$ cDC1 cells and (ii) CD45.1^{+} B6 WT cDC2 cells over CD45.2^{+} *Cxcr5*$^{-/-}$ cDC2 cells. FDR and p values are provided. See Methods for DEG definition utilized in Fig. 4.

Table S2 related to Figure 4. Gene set enrichment analysis (GSEA) results comparing transcriptional profiles of B6 and *Cxcr5*$^{-/-}$ migratory cDC2 cells from msLN of d8 *Hp*-infected mice. GSEA comparing Hallmark gene sets to the rank-ordered list of expressed genes (as defined in Table S1) from B6 wildtype and *Cxcr5*$^{-/-}$ migratory cDC2 cells. Gene sets positively enriched in B6 cDC2 cells are indicated in red font and gene sets positively enriched in *Cxcr5*$^{-/-}$ cDC2 cells are depicted in blue font. The enrichment score (ES), normalized enrichment score (NES), nominal p value (NOM p-val), the FDR q-value and the FWER p-value for each gene set are provided. Gene sets with FDR q-value <0.05 were considered significantly enriched.

Table S3 related to Figure 4. Ingenuity Pathway Analysis (IPA) results comparing transcriptional profiles of B6 and *Cxcr5*$^{-/-}$ migratory cDC2 cells from msLN of d8 *Hp*-infected mice. Genes meeting the threshold of at least 1 RPKM average expression in B6 wildtype or *Cxcr5*$^{-/-}$ cDC2 cells (10,774 genes) AND meeting the FDR q< 0.05 threshold (85 genes, see Table S1) were imported into Ingenuity Pathway Analysis (IPA, QIAGEN Digital Insights) to identify predicted signaling pathways associated with the 85 gene list. Canonical pathways with Benjamin-Hochberg (B-H) corrected p value of <0.05 are listed with $-\log_{10}$ (B-H) corrected p value and z-score. A positive z-score predicts activation of the pathway in B6 wildtype cDC2 cells.

Table S4 related to Figure 4. Ingenuity Pathway Analysis (IPA) results comparing transcriptional profiles of B6 and *Cxcr5*$^{-/-}$ migratory cDC2 cells from msLN of d8 *Hp*-infected mice. Genes meeting the threshold of at least 1 RPKM average expression in B6 wildtype or *Cxcr5*$^{-/-}$ cDC2 cells (10,774 genes) AND meeting the FDR q< 0.05 threshold (85 genes, see Table S1) were imported into Ingenuity Pathway Analysis (IPA, QIAGEN Digital Insights) to identify potential upstream regulators of the 85 genes. Regulators with Benjamin-Hochberg (B-H) corrected p value of <0.05 are included. The type of regulator and targets of the regulator in the dataset are indicated. The absolute and

Benjamin-Hochberg (B-H) corrected p values, and the activation z-score values are provided. A positive z-score predicts activation of the regulator in B6 wildtype cDC2 cells.

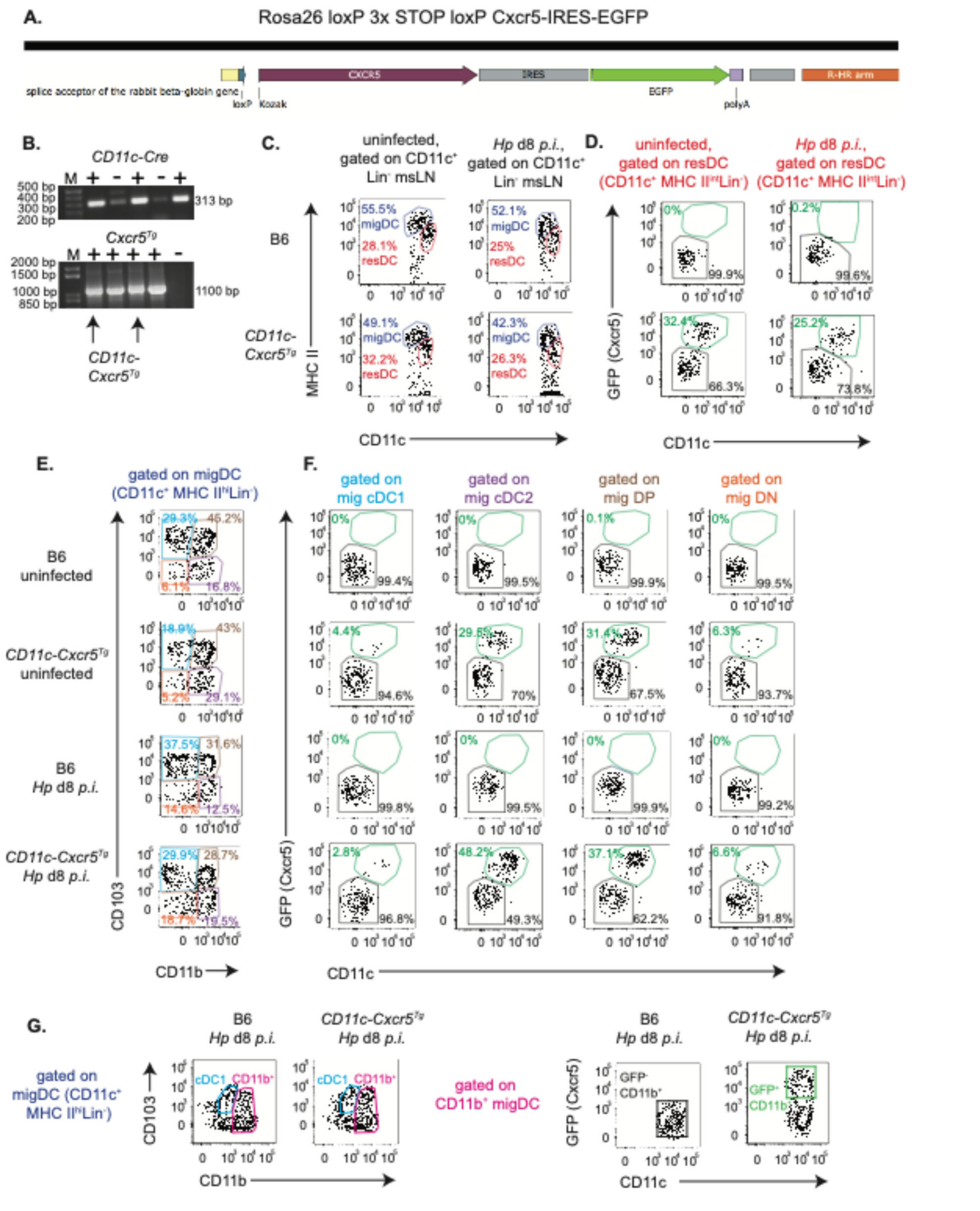

Supplemental Figure S1 related to Figure 3. A CXCR5 transgene, driven by the ROSA26 promoter, is expressed by CD11b⁺ migratory DCs but not CD103⁺ migratory DCs. (**A-B**) Generation of CD11c-*Cxcr5*^{Tg} mice. The ROSA26-loxP-3xSTOP-loxP-CXCR5-IRES-EGFP construct (**A**) was

knocked into the ROSA26 locus in B6 ES cells to generate mice carrying the cre-inducible CXCR5 transgene. The cre-inducible *CXCR5^{Tg}* mice were crossed to *CD11c*-cre mice to generate the *CD11c-CXCR5^{Tg}* strain (**B**). CD11c^{+} cells in these mice express Cre, which removes the STOP codon from the *CXCR5^{Tg}* and allows expression of CXCR5 and the transgene reporter, GFP, under the control of the ubiquitous ROSA26 promoter. (**C-F**) CXCR5 transgene and GFP reporter expression by CD11c^{+} DC subsets in msLNs of uninfected and d8 *Hp*-infected CD11c-*Cxcr5^{Tg}* and B6 mice (n=5-6/group). Representative flow plots depicting CD11c^{+}Lin^{-} (7AAD^{-}CD3^{-}B220^{-}Ly6G^{-}Ly6C^{-}CD64^{-}) conventional DCs (**C**) subdivided into migratory (mig DCs, CD11c^{+}MHCII^{hi}) and resident (res DCs, CD11c^{+}MHCII^{int}) DCs. Representative flow plots depicting subdivision of migratory DC subsets (**E**) into cDC1 (CD103^{+}CD11b^{+}), cDC2 (CD103^{-} CD11b^{+}), DP (CD103^{+}CD11b^{+}), and DN (CD103^{-}CD11b^{-}) cells. Representative flow plots depicting CXCR5 staining and GFP expression on res DCs (**D**) and the different migratory DC subsets (**F**). The numbers of transgene expressing DCs are provided in Fig. 3D-E. (**G**) Sorting strategy to test the T$_{H}$2 priming capacity of CD11b^{+} migratory DCs expressing the CXCR5 transgene. CD11c^{+} cells from msLNs of d8 *Hp*-infected C57BL/6 and *CD11c-Cxcr5^{Tg}* mice (n=5 mice/group) were bead enriched, stained and then sort-purified for *in vitro* T$_{H}$2 priming cultures. The Lin^{-}(7AAD^{-}CD3^{-}B220^{-}NK1.1^{-}Ly6G^{-}Ly6C^{-}CD64^{-}) CD11b-expressing (red gate, includes both DP and cDC2 cells) CD11c^{+}MHCII^{hi} migratory DCs were subdivided into either CXCR5^{-}GFP^{-} DCs (from B6 msLNs) or into the transgene expressing CXCR5^{+}GFP^{+} DCs (from *CD11c-Cxcr5*^{Tg} msLNs). The sorted migratory CD11b^{+} DCs were used in the OTII.4get CD4 T cell priming cultures shown in Figure 3H-I. Data representative of ≥ 3 independent experiments.

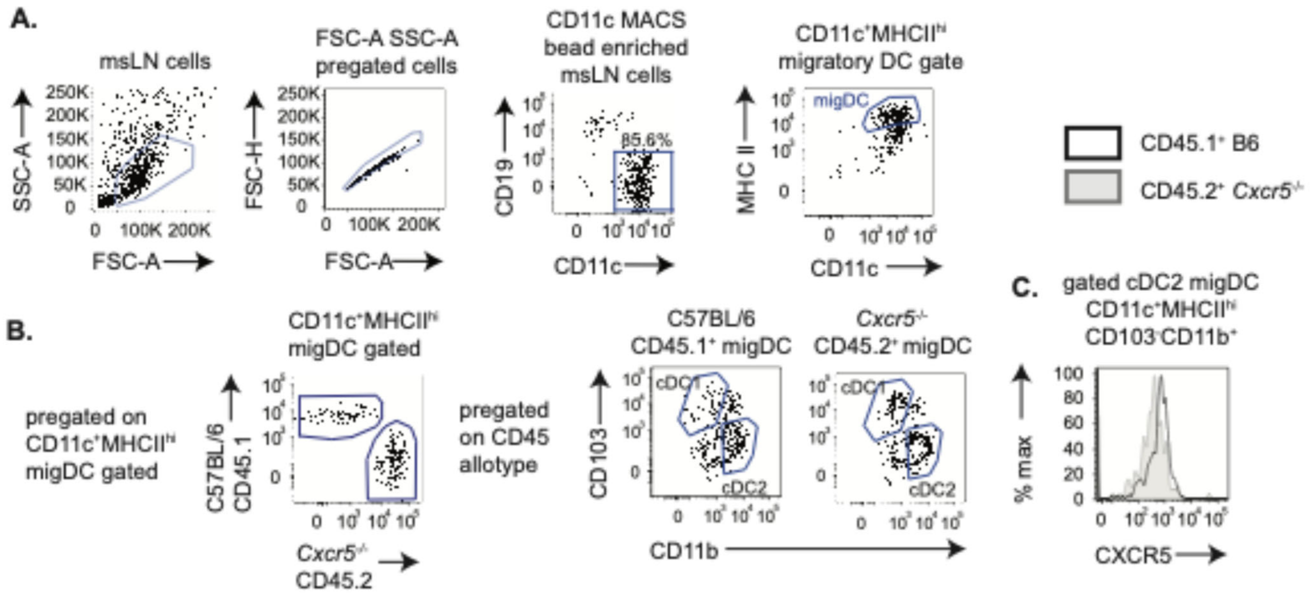

Figure S2 related to Figure 4. Sorting strategies for cDC1 and cDC2 and CXCR5 expression by msLN cDC2 cells from *Hp*-infected msLN. (**A-B**) B6 and *Cxcr5*$^{-/-}$ msLN migratory CD11b^{-}CD103^{+} cDC1 cells and CD11b^{+}CD103^{-} cDC2 cells isolated from *Hp*-infected 1:1 B6:*Cxcr5*$^{-/-}$ mice (n=15). DC were enriched from pooled msLN preps with CD11c beads, subjected to surface staining, and sorted by CD11c+MHCIIhi migratory cDC (Fig. S2A), then by CD45 congenic marker and then into cDC1 (CD11b^{-}CD103^{+}) and cDC2 (CD11b^{+}CD103^{-}) gates (Fig. S2B). Infections and sorts were repeated 3 times, and sorted populations were subjected to paired RNAseq analysis (Fig. 4A-F). CXCR5 expression on sorted B6 and *Cxcr5*$^{-/-}$ cDC2 is shown in Fig. S2C. Statistical analysis for RNAseq datasets (**A-F**) is summarized in Methods.

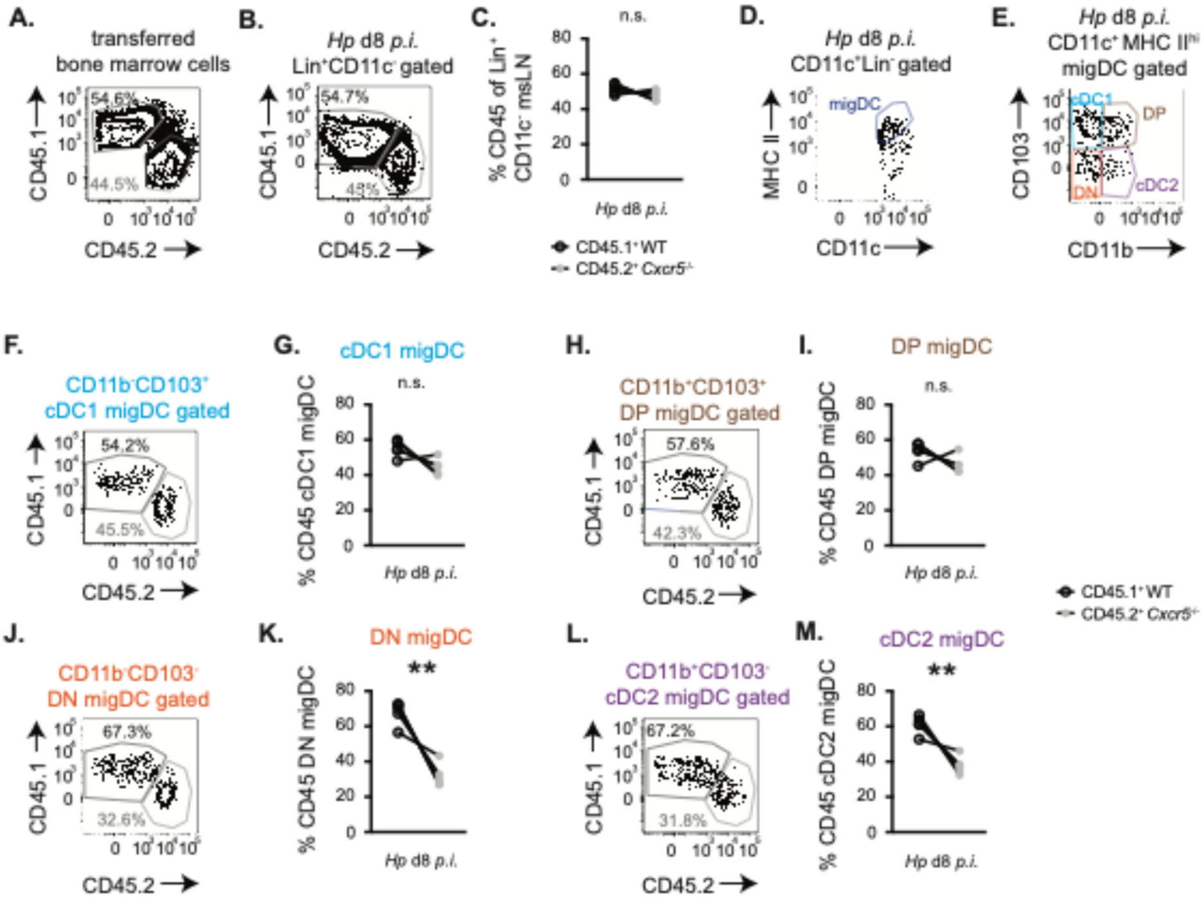

Supplemental Figure S3 related to Figure 4. Effects of DC-intrinsic *Cxcr5* deficiency on the migratory DC compartment in the msLN of *Hp*-infected mice. Analysis of msLN cells in d8 *Hp*-infected 1:1 B6:*Cxcr5*$^{-/-}$ chimeric mice, which were generated as described in Fig. 1A. (**A**) Flow plots showing frequencies of B6 CD45.1^{+} and *Cxcr5*$^{-/-}$ CD45.2^{+} BM cells used to reconstitute irradiated CD45.1^{+} B6 recipients to generate the 1:1 B6:*Cxcr5*$^{-/-}$ chimeric mice. (**B-C**) Analysis of the non-DC leukocytes (CD11c^{-} and positive for one or more lineage markers identifying T, B, NK, or granulocyte) present in the msLN of d8 post-*Hp* infected 1:1 B6:*Cxcr5*$^{-/-}$ chimeric mice. Representative flow plot (**B**) showing CD45.1 and CD45.2 expression by the non-DCs. The percentage of wildtype CD45.1^{+} (open circles) and *Cxcr5*$^{-/-}$ CD45.2^{+} (gray circles) msLN non-DCs (**C**) is reported with the line indicating paired analysis of cells of each genotype present in the same recipient. (**D-M**) Analysis of msLN migratory CD11c^{+}MHCII^{hi} DCs from d8 *Hp*-infected 1:1 B6:*Cxcr5*$^{-/-}$ mice. Representative gating (**D**) of msLN migratory DCs (MHCII^{hi}CD11c^{+}), pregated on Lin^{-} (7AAD^{-}CD3^{-}B220^{-}NK1.1^{-}Ly6G^{-}Ly6C^{-}CD64^{-}) CD11c^{+} cells, which were then subdivided (**E**) using CD11b and CD103 expression into cDC1 (CD103^{+}CD11b^{-}), DP (CD103^{+}CD11b^{+}), DN (CD103^{-}CD11b^{-}), and cDC2 (CD103^{-}CD11b^{+}) cells. Representative flow plots showing CD45.1 and CD45.2 expression by the migratory cDC1 cells (**F**), DP cells (**H**), DN cells (**J**) and cDC2 cells (**L**). The percentage of wildtype CD45.1^{+} (open circles) and *Cxcr5*$^{-/-}$ CD45.2^{+} (gray circles) migratory cDC1 cells (**G**), DP cells (**I**), DN cells (**K**) and cDC2 cells (**M**) is reported with the line indicating paired analysis of cells of each genotype present in the same recipient. The numbers of cells in each migratory DC subset are shown in Fig. 4G-J. Data is representative of at least 3 independent experiments. Statistical analysis was performed with paired 2-tailed student's t-test. Individual wildtype CD45.1^{+} (open circles) and *Cxcr5*$^{-/-}$ CD45.2^{+}

(gray circles) chimeras are shown; pairs are indicated by line graphs. *p≤0.05, **p≤0.01, ***p≤0.001, ****p≤0.0001. n.s. indicates p > 0.05.

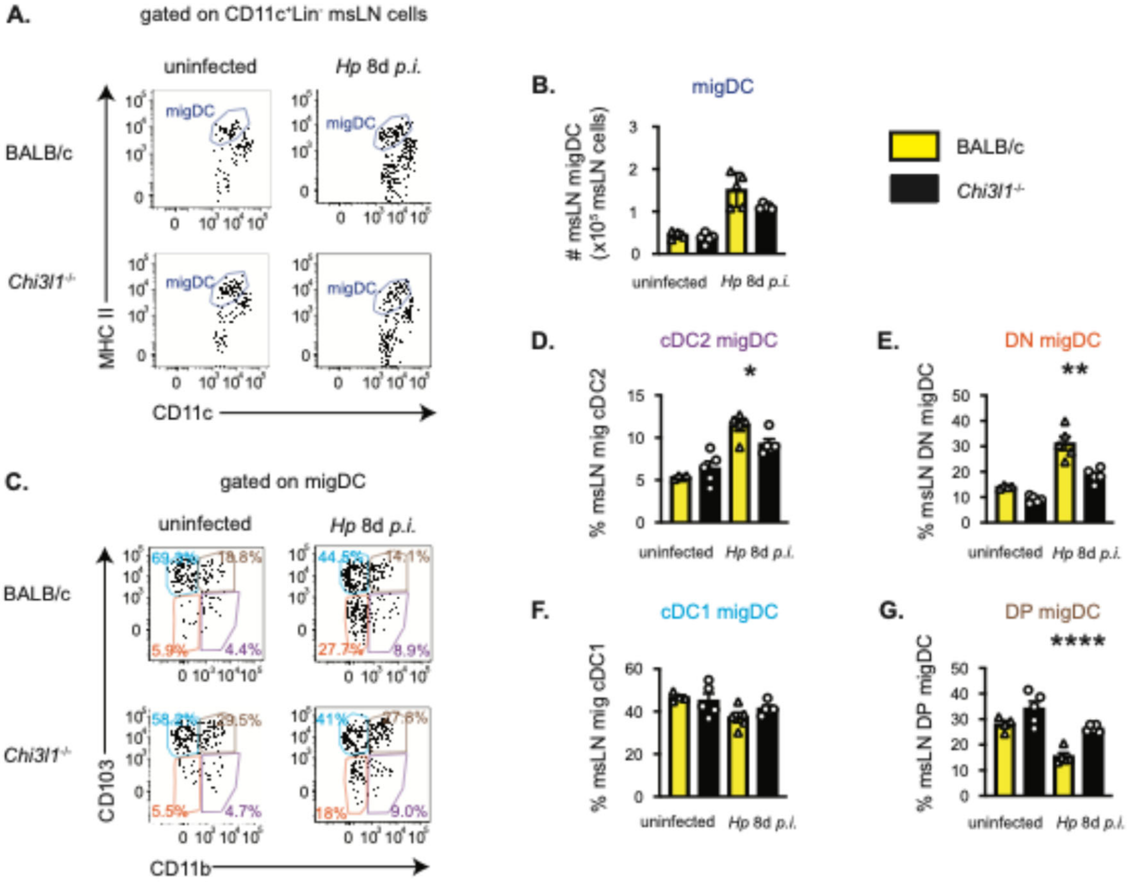

Supplemental Figure S4 related to Figure 5. Global *Chi3l1* deficiency reduces the size of the msLN migratory cDC2 and DN subsets following *Hp* infection. Analysis of migratory DC subsets in msLNs of uninfected and d8 *Hp*-infected BALB/c (yellow bars) and *Chi3l1*$^{-/-}$ (black bars) mice (n=5/group). (**A-B**) Analysis of total CD11c$^+$Lin$^-$ (7AAD$^-$CD3$^-$B220$^-$Ly6G$^-$Ly6C$^-$CD64$^-$) migratory (CD11c$^+$MHCII^{hi}) DC compartment with representative flow plots (**A**) showing the migratory DC population (mig DCs) and bar plots (**B**) showing the number of total migratory DCs in msLNs before and after infection. (**C-G**) Analysis of msLN migratory cDC subsets. Representative flow plots (**C**) depicting gating of migratory cDC subsets and bar graphs showing frequency of the cDC2 (**D**), DN (**E**), cDC1 (**F**), and DP (**G**) cells before and after infection. The number of cells in each migratory DC subset is shown in Fig. 5E-H. Data is representative of at least 3 independent experiments. Statistical analysis was performed with unpaired 2-tailed student's t-test. mean±SD of each group with individual animals depicted as circles or triangles. *p≤0.05, **p≤0.01, ***p≤0.001, ****p≤0.0001. n.s. indicates p > 0.05.
